# Asteroids, ophiuroids and holothurians from the southeastern Weddell Sea (Southern Ocean)

**DOI:** 10.3897/zookeys.434.7622

**Published:** 2014-08-14

**Authors:** Julian Gutt, Dieter Piepenburg, Joachim Voß

**Affiliations:** 1 Alfred Wegener Institute Hemholtz, Centre for Polar and Marine Research, Columbusstraße, 27515, Bremerhaven, Germany; 2 Landesamt für Landwirtschaft, Umwelt und ländliche Räume, Hamburger Chaussee 25, 24220, Flintbek, Germany

**Keywords:** Asterozoa, Asteroidea, Ophiuroidea, Holothuroidea, southern and southeastern Weddell Sea, Antarctic, whole-assemblage approach, abundances, community analysis

## Abstract

Until the early 1980s, the composition and distribution of the asteroid (starfish), ophiuroid (brittle star) and holothurian (sea cucumber) bottom fauna of the southeastern Weddell Sea was virtually unknown. This southernmost part of the Atlantic sector of the Southern Ocean is a typical high-latitude Antarctic region located in the circumpolar permanent pack-ice zone. It became accessible for large-scale scientific surveys only through the availability of modern ice-breaking research vessels, such as the German RV “Polarstern”. Here, we describe a dataset of the faunal composition and abundance of starfish, brittle star and sea cucumber assemblages in this area, based on collections from trawl catches carried out during three “Polarstern” cruises in 1983, 1984 and 1985. The set comprises a total of 4,509 records of abundances of 35 asteroid species (with a total of 2,089 specimens) and 38 ophiuroid species (with a total of 18,484 specimens) from 34 stations, as well as of 66 holothurian species (with a total of 20,918 specimens) from 59 stations including zero-abundances (absences). A synthesizing zoogeographical community analysis confirms the presence of three distinct assemblages of asteroids, ophiuroids, and holothurians with highest species richness on the eastern shelf. Overall, starfishes, brittle stars and sea cucumbers were present at all sites investigated in the study area but composition and abundance of asterozoan (asteroids and ophiuroids together) and holothurian fauna varied considerably. A synthesizing zoogeographical community analysis confirms the presence of three distinct assemblages of asteroids, ophiuroids, and holothurians with highest species richness on the eastern shelf. In the case of asterozoans, water depth and latitude seemed to be the most important drivers of assemblage distribution and composition. One of the holothurian assemblages was part of the rich macrozoobenthic community dominated by a diverse and abundant epifauna, mainly sponges and gorgonians. Another one was mainly composed of vagrant deposit-feeding species inhabiting a predominantly non-colonised substratum. In addition, a mixed holothurian assemblage was identified.

## Introduction

The southeastern Weddell Sea in the Atlantic Sector of the Southern Ocean is a typical high-latitude Antarctic region. It is located in the circumpolar permanent pack-ice zone (Hempel 1985a), characterized by summerly polynyas (areas of open water surrounded by sea ice). Due to its remoteness and persistent sea-ice cover, it was not accessible for extensive scientific surveys before the availability and support of modern research platforms that are capable to operate independently in sea-ice covered waters.

**Figure 1.**
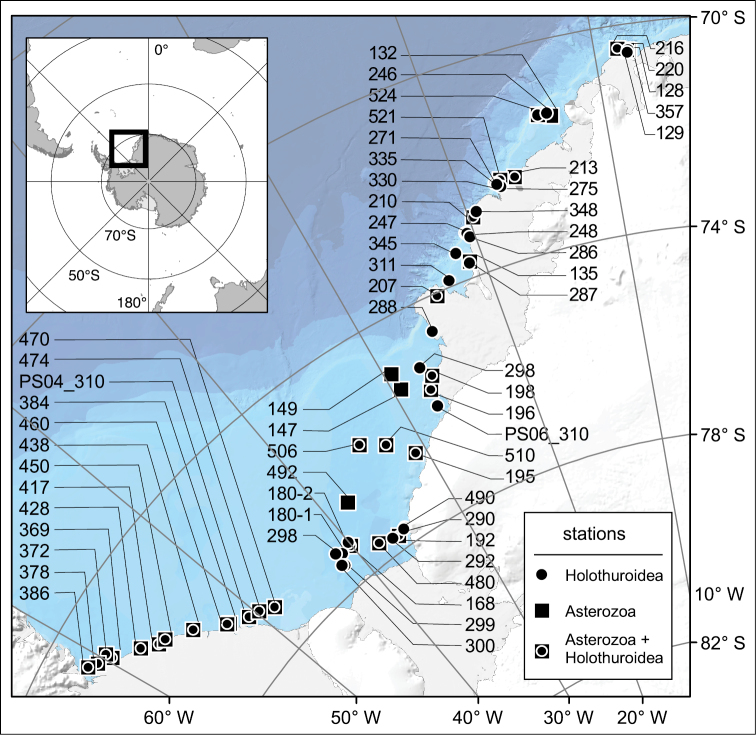
Map of stations in the southeastern Weddell Sea (Southern Ocean) where either asterozoans and holothurians separately or both asterozoans and holothurians together were sampled during „Polarstern“ cruises PS01, PS04, and PS06. In case of station number 310 that occurred during two cruises, cruise numbers are included in the station labels.

The first multidisciplinary marine research was carried out in this area in the 1980s during the first Antarctic cruises of the German icebreaking research and supply vessel "Polarstern”. Embedded in a broad ecological research programme, addressing a range of evolutionary, systematic, zoogeographical and ecological issues, first comprehensive faunistic inventories of the asteroid (starfish), ophiuroid (brittle star) and holothurian (sea cucumber) bottom fauna were conducted, based on field sampling efforts (Fig. 1) during “Polarstern” cruises ANT-I/2 (PS01), ANT-II/04 (PS04), and ANT-III/3 (PS06) (for cruise reports see Hempel 1983, Drescher et al. 1983, Kohnen 1984, and Hempel 1985b, respectively).

The major objective of this collection work was to provide material for subsequent zoogeographical and ecological studies on the asterozoan (asteroid and ophiuroids together; Voß 1988, Piepenburg et al. 1997) and holothurian fauna (Gutt 1988, Gutt 1991), as well as for analyses of entire macrozoobenthos communities (Gutt 2000; for a compilation: Gutt et al. 2013). Here, we publish the complete original dataset of asteroid, ophiuroid and holothurian abundances, including zero-abundances (= absences in the catches) that formed the basis of the scientific findings published in the research papers mentioned above, to allow for the general accessibility to such data associated with starfishes, brittle stars and sea cucumbers from the southeastern Weddell Sea.

In addition to making data and metadata available in the public database ANTABIF (www.biodiversity.aq), a robust community and diversity analysis for holothurians and asterozoans combined was carried out to synthesize results already published for holothurians and asterozoans separately (Voß 1988, Gutt 1991, Piepenburg et al. 1997).

## Study area, material and methods

Asteroid, ophiuroid, and holothurian specimens were sampled at a total of 59 sites distributed across the southeastern Weddell Sea at water depths between 160 and 1,180 m (Fig. 1; for more detailed information see section on “Geographic coverage” below). In general, this region is characterized by a relatively homogenous physical environment, especially in terms of water masses, poorly sorted sediments, persistent sea-ice cover and hardly predictable occurrence of coastal polynyas. As such, it is representative for the entire high-latitude Antarctic habitat. Some drivers of faunistic heterogeneity, in addition to biological interactions and unknown unpredictable factors, are briefly summarized in the section on “Project Data – Study area description” below.

The field samples were mainly taken by means of an Agassiz trawl, but also with a commercial bottom trawl and, in one case, a smaller dredge. During the cruises, GPS positions were available approximately each six hours. Between the GPS fixes, the ship's positions were death reckoned (ship's track calculated by means of ship's speed and course starting from a satellite fix). Swept areas were estimated for each haul as described in Voß (1988) and Gutt (1988). Water depths were measured by a DWD echo sounder. For more detailed information see section on “Sampling methods – Sampling description” below.

Specimens were collected from either total catches or, in some cases subsamples, counted and preserved on board. Using the swept-area estimates, individual counts were standardized to abundance values (ind m^-2^). After the cruises, the preserved specimens were identified to species in the lab. Some holothurian species, which were assumed to be new to science, were formally described (Gutt 1990a, b). Some of these new descriptions were later revised. The specimens were not integrated into a museum's collection, and original data were never published at that time when electronic data bases did not yet exist.

The quality of the data and metadata published here was enhanced prior to publication following the best practices suggested in the literature during the digitalization and geo-referencing processes. Moreover, the current accurate spelling of scientific names – except for the ophiuroid *Theodoria conveniens* ("nomen dubium") – was reviewed based on the World Register of Marine Species (www.marinespecies.org/). For more information see “Sampling methods – Quality control” below.

For 26 stations, at which both holothurians and asterozoans were sampled from Agassiz trawl catches, a simple multivariate community analysis of combined holothurian and asterozoan data were carried out, using the PRIMER 6.1.6 software (Clarke and Warwick 2001, Clarke and Gorley 2006). Abundance values (ind m^-2^) were standardized to percentages per catch, to eliminate bias possibly introduced by differences between-haul catchability. Between-station resemblances were quantified by means of the Bray-Curtis similarity coefficient. The overall pattern of taxonomic resemblances was investigated using cluster analysis (average linkage) and Multidimensional Scaling (MDS).

## Results

The dataset comprises a total of 4,509 records of abundances of 35 asteroid species (with a total of 2,089 specimens) and 38 opiuroid species (with a total of 18,484 specimens) from 34 stations, as well as of 66 holothurian species (with a total of 20,918 specimens) from 59 stations including zero-abundances.

**Figure 2.**
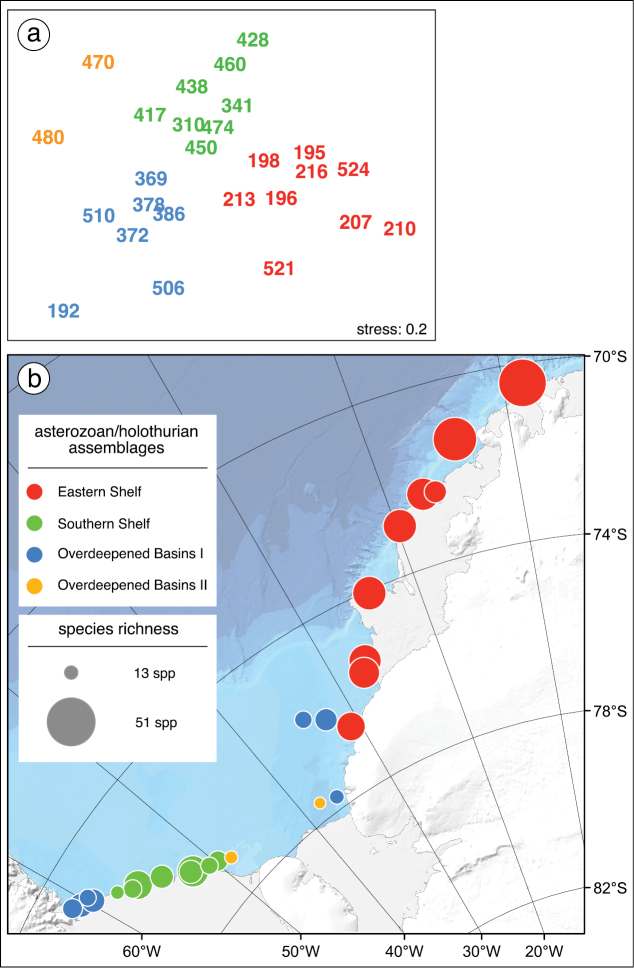
Faunal resemblance pattern (**a**) and geographic distibution (**b**) of stations where both asterozoans and holothurians were sampled during „Polarstern“ cruises PS01, PS04, and PS06. **a** Multidimensional Scaling (MDS) plot showing the faunal between-station resemblance pattern. The numbers are station numbers, the affiliation of stations to asterozoan-holothurian assemblages, based on cluster analysis (complete linkage, threshold of 21% Bray-Curtis similarity), are indicated by color codes. According to cluster analysis, stn 213 belongs to cluster "Overdeepened Basins II" but based on MSD ordination it was assigned to cluster "Eastern Shelf" **b** Geographic map of stations. Symbol color denotes assemblage affiliation according to cluster analysis and Multidimensional Scaling (see Fig. 2a), symbol size is scaled according to the number of species at each station (ranging from 13 to 51).

**Figure 3.**
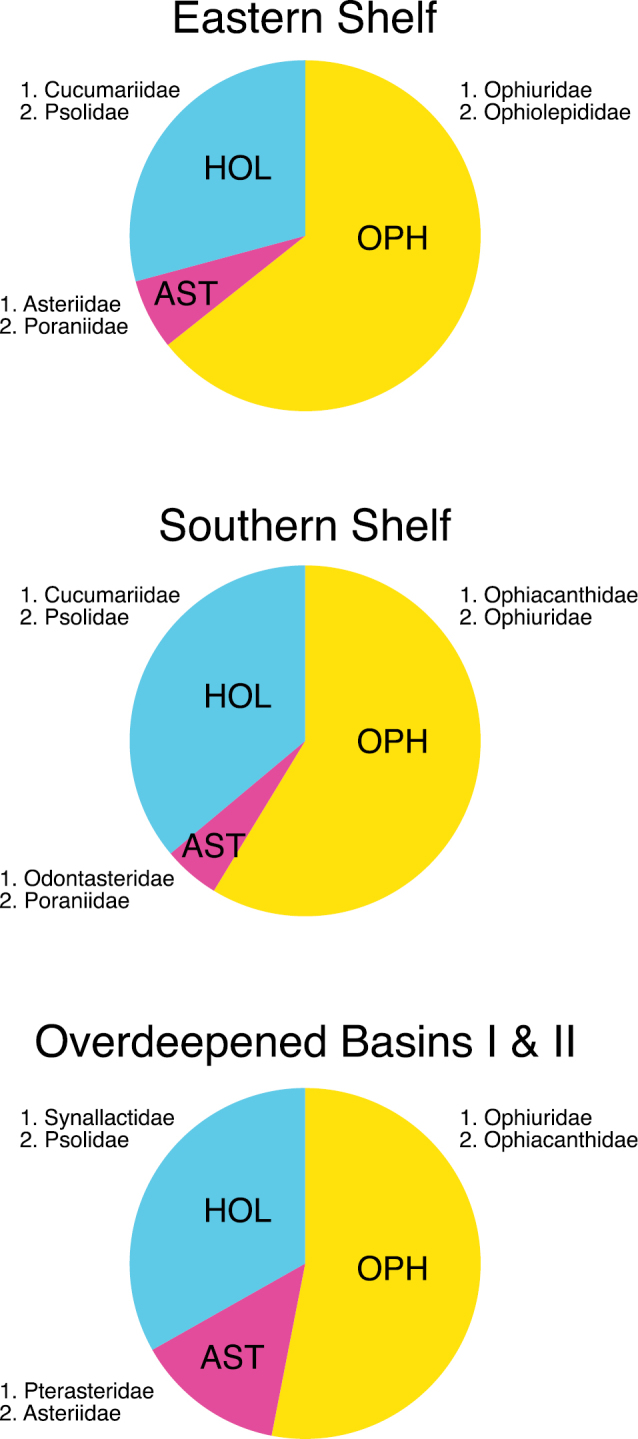
Relative abundance proportions of Asteroidea (AST), Ophiuroidea (OPH) and Holothuroidea (HOL) within the three assemblages (Eastern Shelf, Southern Shelf, and Overdeepened Basins I & II) defined by cluster analysis and multidimensional scaling. In addition, the two most abundant families are given for each echinoderm class.

Asteroid, ophiuroid and holothurian species were present at all sites investigated in the study area but composition and abundance of the asterozoan and holothurian assemblages varied considerably. The synthesizing community analysis shows four holothurian-asterozoan clusters. Since the cluster "Overdeepened Basins II" shows an affinity to "Overdeepened Basins I" rather than to "Eastern Shelf" (Fig. 2a), these two clusters were merged for analyzing the family-level composition. The "Eastern Shelf" assemblage was richest in species, "Overdeepened Basins II" was poorest, and the others were similar to each other, with intermediate species numbers (Fig. 2b). The class-level relative abundances were similar in all clusters, with highest values for ophiuroids followed by holothurians and lowest for asteroids (Fig. 3). However, on a family level, major differences became visible between the assemblages, whilst in the "Overdeepened Basins I & II" deposit-feeding holothurians (Synallactidae) were dominant. Filter feeders (Psolidae and Cucumariidae) were most abundant in "Eastern Shelf" and "Southern Shelf". Among asteroids, *Hymenaster* spp., of the family Pterasteridae, a typical deep-sea form, were most abundant at basin sites at water depths down to almost 1,200 m. The genus *Odontaster*, being generally abundant in the Antarctic, was dominant in the assemblage "Southern Shelf"; apart from that the evenness among the asteroids was more obvious than among holothurians and ophiuroids. A major difference between ophiuroid assemblages was the second dominance of Ophiolepididae in the "Eastern Shelf" assemblage, with *Ophioceres incipiens* being generally rare but occurring locally in relatively high abundances. It is a diatom feeder (Dahm 1996) and the smallest species in the entire area. The obvious high abundance of Ophiuridae across all clusters is due to the dominance of various species: the omnivorous large-sized *Ophionotus victoriae* in the assemblage "Overdeepened basins II", the crustacean feeder *Ophioplinus gelida* in "Southern Shelf" and the shallower shelf prefering *Ophioplinthus martensi* and the deeper *Ophioplinthus brevirima* in "Eastern Shelf". Since *Ophiacantha antarctica* was by far the most abundant ophiuroid in "Southern Shelf" Ophiacanthidae was the dominant family in this assemblage.

In the case of the separate analysis of asterozoan patterns (Voß 1988, Piepenburg et al. 1997), water depth and latitude seemed to be the most important drivers of assemblage distribution and composition. At shallow shelf sites rare asteroid and ophiuroid predators, such as, e.g., *Acodontaster conspicuus* and *Odontaster validus*, respectively, as well as epibiotic ophiuroids, e.g., *Astrotoma agassizii*, occurred in addition to the common and widely distributed opportunistic feeders. In the case of the separate analysis of holothurians (Gutt 1988, Gutt 1991), one assemblage co-occurred with the rich macrozoobenthic community dominated by dense epifauna consisting of, e.g., sponges and gorgonians, which are often used as substratum by epibiotic filter feeders. Others live mainly as vagrant deposit feeders on the predominantly non-colonised substratum, such as typical deep-sea species, e.g., *Elpidia glacialis* and *Protelpidia murrayi* (Gutt and Piepenburg 1991). In addition, a mixed holothurian assemblage was identified.

## General significance

Virtually nothing was known about echinoderms in the southeastern Weddell Sea before the field sampling work, in the course of which the data published here was recorded. Also from other Antarctic regions only sporadic information on the three echinoderm classes, especially holothurians, was available at that time, mainly found published in the taxonomic literature. However, the application of a whole-assemblage approach was novel, and comparable surveys are even nowadays rare.

The unique dataset encompasses some of the first observations of asteroids, ophiuroids and holothurians in this area and represents a significant contribution of primary data about Antarctic benthos assemblages. Moreover, it provides unique baseline data for future faunistic, ecological and conservation studies to evaluate the effects of climate change and possible future fishing activities in this area. At present and in the future these faunistic data can gain further importance in the context of a number of further applications:

(1) More comprehensive circumpolar analyses and comparative studies with other large taxa become possible if these data are merged with similar datasets from other regions, see e.g., http://ipt.biodiversity.aq/resource.do?r=asteroidea_zoogeography and http://ipt.biodiversity.aq/resource.do?r=biopearl_asteroida. Data on these three echinoderm classes can also be compared within the same region with other taxa or environmental parameters, as compiled, e.g., by De Broyer et al. (2014).

(2) At the time of sampling in the 1980s, the study area was almost pristine and hardly affected by any anthropogenic activities. In the meantime, exploratory fishing started on the deeper shelf, and the knowledge on the fauna before the onset of these activities can serve as a valuable baseline for an assessment of the impact of further fishing.

(3) The same holds true for the study of the effects of climate change. The area is so far climatologically relatively stable but an increase of bottom-water temperatures is expected during the 21st century (Hellmer et al. 2012).

(4) The data can also be used for nature conservation initiatives (Teschke et al. 2013). They are especially suited for such applied ecological comparative studies, since all specimens – with few well-defined exceptions – were sorted from the catches, meaning that not only the presence of the species were registered but also very valuable absence data and the even more informative abundances. In general, the latter data are known to be more sensitive indicators of environmental change than binary presence-absence data.

(5) The community analysis of the combined holothurian-asterozoan data largely confirms the findings of the previous separate analyses of holothurians and asterozoans (Voß 1988, Gutt 1991, Piepenburg et al. 1997). There are basically three assemblages, the composition of which are quite similar on the level of classes but strongly differing on the level of families and species, as well as with regard to trophic guilds. These resemblance patterns can be attributed to geographic and bathymetric conditions.

## General description

**Purpose:** The publication of the complete dataset of asteroid, ophiuroid and holothurian abundances (and absences) in the southeastern Weddell Sea, which formed the basis of the scientific findings already published in a number of original research papers, shall allow for the general accessibility to such data associated with starfishes, brittle stars and sea cucumbers from this high-Antarctic region. The unique dataset encompasses some of the first observations of asteroids, ophiuroids and holothurians in the study area and represents a significant contribution of primary data about Antarctic benthos assemblages. Moreover, it provides unique baseline data for future faunistic, ecological and conservation studies to evaluate the effects of climate change and possible future fishing activities in this area.

## Project details

**Project title:** Asteroids, ophiuroids and holothurians from the southeastern Weddell Sea (Southern Ocean)

**Funding:** The sampling of all asterozoan and holothurian specimens in the course of the cruises ANT-I/2 (PS01), ANT-II/04 (PS04), ANT-III/3 (PS06) of the German R/V "Polarstern" and the subsequent analysis of asteroids and ophiuroids was financed by the Alfred Wegener Institute Helmholtz, Centre for Polar and Marine Research, Bremerhaven, Germany. The study on holothurians was also supported by a grant of the Deutsche Forschungsgemeinschaft (He 89/49).

**Study area descriptions/descriptor:** The study area included regions characterized by almost permanent pack-ice cover in the southernmost Weddell Sea as well as regions featuring coastal polynyas in the eastern Weddell Sea (Gutt 2000). The shelf figau is mostly rather narrow, only a few 10 km wide, in some areas even "disappearing" beneath the floating ice shelf, but can also be much broader in the southern Weddell Sea (Arndt et al. 2013). Consequently, near-coast habitats can be affected by larger and smaller floating ice shelves or by a glaciated coast. As there is no "true" (i.e., non-glaciated) coast, shallow littoral habitats (<50 m water depth) are not known from this area.

The shelf seabed is usually rather level, and especially habitats on banks and their flanks are disturbed with varying intensity by grounding or scouring icebergs (Gutt and Starmans 2001). In addition to the shelf below 160 m water depth sampling included the upper slope and stations in the Filcher depression, an overdeepened trough with water depths of up to 1,180 m.

The hydrography of the study area is characterized by a southwestward flowing coastal current, which is part of the large Weddell Gyre (Fahrbach et al. 1992) and flows with a velocity of up to 0.14 m/s above the shelf edge. Different water masses dominated by the "Eastern Shelf Water" close to the sea floor are mainly characterized by low temperatures close to the freezing point and high salinities. However, occasionally upwelling "Warm Deep Water", with an average temperature of 0.4 °C, can be found on the deeper shelf (Fahrbach et al. 1992, Schröder and Fahrbach 1999). In the South, the current regime shows water flow from beneath and under the Filchner-Ronne Ice Shelf, with both northward and southward directions (Grosfeld et al. 2001). This difference is potentially of high relevance for the food supply to the benthos and also shapes species compositions along the eastern coast with smaller ice shelves.

Surface sediments are generally poorly sorted. However, clear differences in the sand-silt proportion exist, with mainly soft sediments in the deep areas and coarser sediments on the shelf. In some areas, biogenic particles, such as bryozoan debris and sponge spicules, are important components of the sediments, sometimes forming dense mat-like structures (Voß 1988).

**Design description:** Asteroid, ophiuroid, and holothurian specimens were sampled during the cruises ANT-I/2 (PS01), ANT-II/04 (PS04), ANT-III/3 (PS06) of the German R/V "Polarstern" at a total of 59 sites distributed across the southeastern Weddell Sea at water depths between 160 and 1,180 m (for more detailed information see section on “Geographic coverage”). The field samples were mainly taken by means of an Agassiz trawl, but also with a commercial bottom trawl and, in one case, a smaller dredge (for more detailed information see section on “Sampling methods - Sampling description”). During the cruises, GPS positions were recorded approximately every six hours. Between the GPS fixes, the ship's positions were death reckoned. Water depths were measured by a DWD echo sounder. Specimens were collected from either total catches or, in some cases subsamples, counted and preserved on board. They were later identified in the lab. Some holothurian species, which were assumed to be new to science, were formally described. Some of these new descriptions were later revised. The specimens were not integrated into a museum's collection, and original data were never published at that time when electronic data bases did not yet exist. The quality of the data and metadata published here was enhanced prior to publication following the best practices suggested in the literature during the digitalization and geo-referencing processes.

## Taxonomic coverage

**General taxonomic coverage description:** All asteroids, ophiuroids, and holothurians caught by the gear mentioned in the section on “Sampling Methods" were considered in this study, with the exception of the very rare species *Amphiura deficiens* Koehler, 1992 and *Amphiura atlantica* Ljungman, 1867. The taxonomic and morphological range even covers two holothurian species, which are assumed or known to be able to swim occasionally, *Rhipidothuria racovitzai* and *Peniagone vignioni*. Due to the mesh size used, not only adult but also juvenile specimens of all three classes are included in the collections. However, their abundance values are likely more biased than those of the adults. The trawls predominantly caught epifaunal species in a semi-quantitative way, the Agassiz trawl obviously with a higher catchability of macro-epibenthic invertebrates than the bottom trawl. Therefore, the swept-area approach is most useful for within-gear comparisons and only with less precision between-gear. The presence of typical infaunal species in the catches, such as the holothurian *Molpadia* and the asteroid *Hymenaster*, suggests that endobenthic species were also sampled to a considerable degree. Overall, organisms from a broad variety of ecological guilds among all three classes, such as deposit, sediment and filter feeders, infaunal, epifaunal and epibiotic (symbiotic) species, predators spezialized on various prey items, and scavengers, are present in the samples.

## Taxonomic ranks

Class: Asteroidea; species: *Bathybiaster loripes*, *Macroptychaster accrescens*, *Leptychaster flexuosus*, *Psilaster charcoti*, *Cheiraster (Luidiaster) gerlachei*, *Acodontaster capitatus*, *Acodontaster conspicuus*, *Acodontaster hodgsoni* f. *hodgsoni*, *Acodontaster hodgsoni* f. *stellatus*, *Acodontaster marginatus*, *Odontaster meridionalis*, *Chitonaster johannae*, *Notioceramus anomalus*, *Cycethra verrucosa*, *Perknaster aurorae*, *Perknaster sladeni*, *Porania (Porania) antarctica*, *Kampylaster incurvatus*, *Pteraster affinis*, *Pteraster stellifer*, *Hymenaster* spp., *Peribolaster macleani*, *Remaster gourdoni*, *Solaster regularis*, *Lophaster densus*, *Lophaster gaini*, *Lophaster tenuis*, *Paralophaster antarcticus*, *Paralophaster godfroyi*, *Paralophaster* sp., *Cuenotaster involutus*, *Henricia parva*, *Henricia smilax*, *Rhopiella hirsuta*, *Diplasterias brucei*, *Kenrickaster pedicellaris*, *Lysasterias digitata*, *Lysasterias perrieri*, *Notasterias armata*, *Notasterias bongraini*, *Notasterias haswelli*, *Notasterias stolophora*, *Pedicellaster hypernotius*, *Psalidaster mordax*.

Class: Ophiuroidea; species: *Astrotoma agassizii*, *Astrochlamys bruneus*, *Ophiacantha antarctica*, *Ophiacantha vivipara*, *Ophiacantha pentactis*, *Ophiocamax drygalskii*, *Ophiomitrella ingrata*, *Ophiomitrella* sp., *Ophiosparte gigas*, *Ophiolimna antarctica*, *Amphiura belgicae*, *Amphiura joubini*, *Amphiura proposita*, *Ophioleuce regulare*, *Ophioceres incipiens*, *Ophiocten dubium*, *Ophiocten doederleini*, *Ophiocten megaloplax*, *Ophionotus victoriae*, *Ophioperla koehleri*, *Ophioplinthus brucei*, *Ophiosteira debitor*, *Ophiosteira echinulata*, *Ophiosteira rotundata*, *Ophiura lymani*, *Ophiura (Ophiuroglypha) carinifera*, *Ophioplinthus brevirima*, *Ophioplinthus gelida*, *Ophioplinthus martensi*, *Ophioplinthus tumescens*, *Ophiogona doederleini*, *Ophiura flexibilis*, *Ophiura (Ophiuroglypha) irrorata*, *Ophiura rouchi*, *Theodoria conveniens*, *Ophioplinthus relegata*, *Anophiura* sp., *Amphiophiura* sp.

Class: Holothuroidea; species: *Cucumaria georgiana* s.l., *Psolidiella mollis*, *Cucamba psolidiformis*, *Microchoerus splendidus*, *Trachythyone parva*, *Trachythyone bouvetensis*, *Staurocucumis liouvillei*, *Staurocucumis turqueti*, *Heterocucumis steineni*, *Heterocucumis denticulata*, *Paracucumis turricata*, *Crucella scotiae*, *Crucella hystrix*, *Psolus dubiosus*, *Psolus charcoti*, *Psolus antarcticus*, *Psolicrux coatsi*, *Psolidium gaini*, *Psolidium poriferum*, *Echinopsolus acanthocola*, *Bathyplotes moseleyi* s.l., *Bathyplotes gourdoni*, *Bathyplotes bongraini*, *Pseudostichopus mollis*, *Molpadiodemas villosus*, *Mesothuria (Zygothuria) lactea*, *Laetmogone wyvillethomsoni*, *Rhipidothuria racovitzai*, *Peniagone vignoni*, *Protelpidia murrayi*, *Sigmodota contorta*, *Paradota weddellensis*, *Molpadia musculus*.

**Common names:** Starfish, Brittle stars, Sea cucumbers.

## Spatial coverage

**General spatial coverage:** The study area extends northward to 70°27'S, a latitude which is typical for the northern shelf off East Antarctica, with the exception of the large embayments of the Weddell and Ross Seas, and for the more southerly situated West Antarctic shelf regions, with the exception of the Antarctic Peninsula area. The southernmost station in the study area was located at 77°44'S. With regard to its longitudinal extent, the study area ranged from 008°01'W in the eastern Weddell Sea to 061°08'W at the basis of the Antarctic Peninsula at 061°08'W.

With regard to water depth, the samples were taken along a gradient ranging from 160 m at the shelf to a maximum of 1,180 m at the upper slope, encompassing stations at relatively shallow banks as well as those in overdeepened basins, such as the Filchner Trough.

**Coordinates:** 78°0'0"S and 70°0'0"S Latitude; 62°0'0"W and 8°0'0"W Longitude.

**Temporal coverage:** February 4, 1983 – February 24, 1985.

## Methods

**Method step description:** See "Sampling description".

**Study extent description:** See "Study area description".

**Sampling description:** Three sampling gears were used: An Agassiz trawl that was deployed most frequently consisted of a metal sled, with an opening of 3 m width and 1 m height, to which a net, which was 5 m long and had a mesh size of 20 mm in the front part and 10 mm in the cod end, was attached. In front of the opening a tickler chain was fixed to the rig of the sled. The average trawling speed was 0.5 to 0.7 knots (nm/h), and the haul duration was approx. 20-30 min, resulting in swept areas of approx. 1,700 to 3,000 m^2^. This strategy was a compromise to gain comparable semi-quantitative information about both relatively poor Antarctic communities, based on catches that were, nevertheless, large enough to be representative, and rich communities without clocking the net.

The bottom trawl used was a 140 feet commercial otter trawl with a 22.5 m wide and approximately 3 m high opening. The mesh size of the net was 10 cm in the front part and 15 mm in the cod end. The bottom trawl was towed over ground at an average speed of 3 knots (nm/h), mostly for 0.5 hours, as it is standard for research fishing of demersal fish.

The dredge used has an opening of 100 × 30 cm and a mesh size of 10 mm. The swept areas were calculated according to Voß (1988) for the asterozoan study and according to Gutt (1988) for the holothurian study.

Due to technical constraints, only one position and time was provided for the catches and only in some cases information on the depths at the start and end of the hauls was available. Metadata (position and time) are available at www.pangaea.de for the expeditions PS01 and PS06. For cruise PS04, metadata were published by Voß (1988), with the exception of stn 490 (Gutt 1988). Metadata are based on the station lists published in the cruise reports (Hempel 1983, Drescher et al. 1983, Kohnen 1984).

**Quality control description:** Identifications were made by Joachim Voss under supervision of Ilse Bartsch for ophiuroids and by Julian Gutt for holothurians partly under supervision of David Pawson (comparison with material at the Smithsonian Institution, Washington) and Bent Hansen (Elasipodida caught during the Galathea expedition). In addition, identifications were based on the taxonomic references cited by Voß (1988) and Gutt (1988). All species names in the dataset are in accordance with the World Register of Marine Species (www.marinespecies.org/), with the sole exception of the ophiuroid *Theodoria conveniens* ("nomen dubium"). Consequently, some species names have changed in comparison to the ones used in past publications, as these are synonyms that are not valid anymore.

## Datasets

### Dataset description

**Object name:** Darwin Core Archive Asteroids, ophiuroids and holothurians from the southeastern Weddell Sea (Southern Ocean)

**Character encoding:** UTF-8

**Format name:** Darwin Core Archive format

**Format version:** 1.0

**Distribution:** http://ipt.biodiversity.aq/archive.do?r=asteroids_and_ophiuroids_from_the_southeastern_weddell_sea

**Publication date of data:** 2014-02-20

**Language:** English

**Licenses of use:** This work is licensed under a Creative Commons CCZero 1.0 License http://creativecommons.org/publicdomain/zero/1.0/legalcode

**Metadata language:** English

**Date of metadata creation:** 2014-01-09

**Hierarchy level:** Dataset

**Metadata language:** English

**Date of metadata creation:** 2014-01-09

**Hierarchy level:** Dataset
